# Bandgap engineering of a lead-free defect perovskite Cs_3_Bi_2_I_9_ through trivalent doping of Ru^3+^†

**DOI:** 10.1039/c8ra04422h

**Published:** 2018-07-18

**Authors:** Jinyu Gu, Gangbin Yan, Yuebin Lian, Qiaoqiao Mu, Huidong Jin, Zaichao Zhang, Zhao Deng, Yang Peng

**Affiliations:** Soochow Institute for Energy and Materials Innovations, College of Physics, Optoelectronics and Energy, Collaborative Innovation Center of Suzhou Nano Science and Technology, Soochow University Suzhou 215006 P. R. China zdeng@suda.edu.cn ypeng@suda.edu.cn; Key Laboratory of Advanced Carbon Materials and Wearable Energy Technologies of Jiangsu Province, Soochow University Suzhou 215006 P. R. China; Jiangsu Key Laboratory for the Chemistry of Low-dimensional Materials, School of Chemistry and Chemical Engineering, Huaiyin Normal University Huai'an 223000 P. R. China

## Abstract

Inorganic defect halide compounds such as Cs_3_Bi_2_I_9_ have been regarded as promising alternatives to overcome the instability and toxicity issues of conventional perovskite solar cells. However, their wide indirect bandgaps and deep defect states severely limit their photoelectronic conversion efficiency when implemented in devices. Trivalent cation substitution has been proposed by previous calculations allowing the engineering of their band structures, but experimental evidences are still lacking. Herein we use the trivalent cation Ru^3+^ to partially replace Bi^3+^ in Cs_3_Bi_2_I_9_, and reveal their structural and optoelectronic properties, as well as the environmental stability. The Ru-doped Cs_3_Bi_2_I_9_ shows a decreasing bandgap with the increasing doping levels and an overall up-shift of band structure, owing to the dopant-induced defect states and thus enhanced phonon–electron coupling. As a result, upon Ru^3+^ doping, the narrowed bandgap and the upward shift of the band structures might facilitate and broaden their applications in optoelectronic devices.

## Abstract 2

Inorganic defect halide compounds such as Cs_3_Bi_2_I_9_ have been regarded as promising alternatives to overcome the instability and toxicity issues of conventional perovskite solar cells.

## Graphical abstract

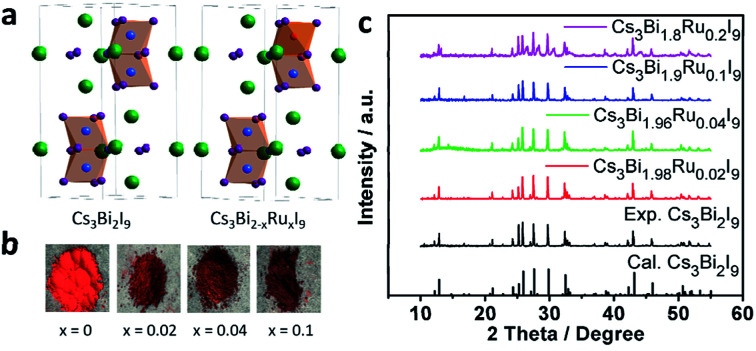

## 1 Introduction

Recently perovskite materials have attracted great attention for their promising applications in optoelectronic devices such as solar cells, light emitting diodes, and photodetectors.^1–6^ In particular, the photoelectron conversion efficiency (PCE) of hybrid organic–inorganic perovskite solar cells (PSCs) has increased rapidly since 2009,^7^ reaching more than 22% up to date.^8^ However, the toxicity and instability of these conventional alkylamine-based PSCs have greatly restricted their larger scale production outside laboratories. In this context, photoabsorbers based on inorganic lead-free perovskites have been pursued as potential alternatives, including three major families: AM^II^X_3_,^9,10^ A_2_M^I^M^III^X_6_ ^11–13^ and A_3_M^III^_2_□X_9_^14,15^ (A = Cs, Rb; M^II^ = Sn, Ge; M^I^ = Ag, Na, K; M^III^ = Bi, Sb;□ = vacancy sites; X = Cl, Br, I), each representing the halide perovskites, double halide perovskites and defect halide perovskites, respectively. With the choice of different elements for A, M and X, the bandgap of these compounds can be effectively tuned.^16^ Further in each of the above perovskite families, the metal M of various chemical states can be further substituted with alloys containing multiple metals with the same total oxidation state (so-called cation transmutation),^17,18^ adding an additional degree of freedom to regulate their bandgaps.

In the defect halide perovskite family A_3_M^III^_2_□X_9_, Cs_3_Bi_2_I_9_ has been mostly studied with the consideration of Bi^3+^ having similar electronic structure and ion radius to Pb^2+^, but much less toxicity.^19–21^ At room temperature, Cs_3_Bi_2_I_9_ exhibits a distorted and defect-modulated hexagonal perovskite structure, in which a pair of [BiI_6_]^3−^ octahedral share faces to form a [Bi_2_I_9_]^3−^ bioctahedra, and every third layer of the octahedral Bi sites is depleted for charge neutrality, resulting in a zero-dimensional (0D) molecular salt crystal structure (space group *P*6_3_/*mmc*).^22^ The 0D structure with separated [Bi_2_I_9_]^3−^ bioctahedra leads to low carrier mobility and therefore poor charge transport, which might be more beneficial for optical properties, rather than photovoltaic applications.^23–25^ For instance, colloidal Cs_3_Bi_2_I_9_ nanocrystals have shown unique absorption spectra arising from the 0D crystal structure, with a very high excitonic binding energy of 300 meV.^23^ Cs_3_Bi_2_I_9_ possesses an average indirect optical bandgap of ∼2 eV, with variations reported from different studies.^24^ DFT calculations indicated a higher effective mass of charge carriers and prominent deep-level defects for Cs_3_Bi_2_I_9_ when compared with their Pb counterparts, and pointed out that defect passivation through external doping or nonequilibrium synthesis might lead to better photovoltaic performance.^25^ Solar cells with Cs_3_Bi_2_I_9_ as the photoabsorber has shown a power conversion efficiency over 1% with enhanced chemical stability and additional room for improvement.^27^ Hence, in order to further promote the performance of Cs_3_Bi_2_I_9_ based optoelectronic devices, bandgap engineering is essential to narrow the optical bandgap, tune the defect chemistry and optimize the electronic structure.^16,28,29^ For example, DFT studies by Hong *et al.* inferred that the bandgap of Cs_3_Bi_2_I_9_ can be reduced by applying dual trivalent metal cations to form Cs_3_BiM^III^I_9_,^18^ although there has been no experimental evidence in this regard.

Discovered since the 1980s', Cs_3_Ru_2_Cl_9_ is another compound of the A_3_M^III^_2_□X_9_ family that also belongs to the *P*6_3_/*mmc* space group with bioctahedra [Ru_2_Cl_9_]^3−^.^30,31^ Inspired by this fact, herein we attempt to dope the Cs_3_Bi_2_I_9_ with various concentrations of Ru^3+^ through atomic substitution of Bi^3+^ in order to regulate its bandgap and optical properties. As a result, crystals of Cs_3_Bi_2−*x*_Ru_*x*_I_9_ with dimensions from several to hundreds of micrometers were obtained by a hydrothermal approach. Through changing the molar ratio of RuCl_3_ and BiI_3_, various doping levels of Ru^3+^ were realized without altering the crystal structure. Measurements using Raman, UV-Vis, Photoluminescence and Ultraviolet Photoelectron Spectroscopy illustrated that even with a small doping amount, the band structure and optical properties of Cs_3_Bi_2−*x*_Ru_*x*_I_9_ can change significantly. Consequently, through effective tuning of the bandgap, defect states and electronic structure, Ru-doped Cs_3_Bi_2_I_9_ perovskites exhibit a great perspective for future optoelectronic applications.

## 2 Experimental section

### 2.1 Materials and preparation

All chemicals were purchased from the Aladdin Company (China) with purity above 99.95% and used without further purification.

#### Cs_3_Bi_2_I_9_

Microcrystals of Cs_3_Bi_2_I_9_ were synthesized by the hydrothermal method. In a 50 mL reactor, 1.5 mmol CsI and 1 mmol BiI_3_ were dissolved in 20 mL concentrated HI acid. The reactor was then sealed and placed into a thermal oven with the temperature hold at 120 °C. After 6 hours, the temperature was turned down at 10 °C/30 min to the room temperature. Powders of red colour were precipitated from the precursor solution. The solids were then filtered out, washed repeatedly with ethanol and diluted HI, and finally dried under vacuum at 60 °C for overnight.

#### Cs_3_Bi_2−*x*_Ru_*x*_I_9_ (*x* = 0.02, 0.04, 0.1, 0.2)

Microcrystals of Cs_3_Bi_2−*x*_Ru_*x*_I_9_ were also synthesized by the hydrothermal method. 1.5 mmol CsI, *a* mmol BiI_3_ and *b* mmol RuCl_3_ (*a* + *b* = 2) were dissolved in 20 mL concentrated HI acid in a 50 mL reactor. The reactor was then sealed and placed into a thermal oven at 120 °C for 6 h. After cooling down, powders of dark red colour were precipitated from the precursor solution. The solids were then filtered out, washed repeatedly with ethanol and diluted HI, and finally dried under vacuum at 60 °C for overnight.

### 2.2 Characterization

The powder X-ray diffraction (XRD) measurements were conducted using a D8 Advance diffractometer (Bruker Corporation, Germany) operating at 40 kV and 40 mA with Cu Kα radiation (*λ* = 1.5406 Å), and the scanning rate was fixed at 4° min^−1^. Scanning electron microscopy (SEM) images and Energy Dispersive X-ray spectroscopy (EDX) were taken by a FEI Scios Microscope (Thermo Fisher, USA). Transmission electron microscope (TEM) images and elemental mapping were taken by the FEI 200 kV G20 Microscope (Thermo Fisher, USA). X-ray photoelectron spectroscopy (XPS) and Ultraviolet Photoelectron Spectroscopy (UPS) were performed on an Escalab 250Xi (Thermo Fisher, USA). For UPS measurements, pristine Au was used to calibrate the Fermi level. Raman spectra were obtained on a Jobin Yvon HR Evolution Spectrometer (Horiba, Japan). Fluorescence Spectroscopy was taken with a FLS980 Fluorescence Spectrometer (Edinburgh instruments, UK). Thermogravimetric analysis (TGA) was conducted with the SII TG/DTA7200 (Japan) at a rate of 20 °C min^−1^ ramping from room temperature to 700 °C inside a crucible. UV-Vis spectroscopy was recorded with PerkinElmer Lambda 750S (USA).

## 3 Results and discussion

### 3.1 Crystalline phase and morphology

**Fig. 1:**
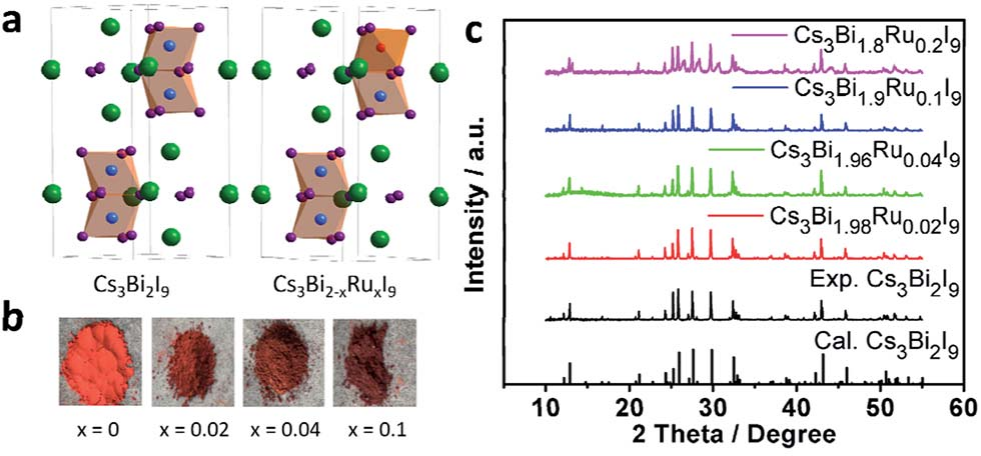
(a) Crystal structure of Cs_3_Bi_2_I_9_ and Cs_3_Bi_2−*x*_Ru_*x*_I_9_. (b) Photos of samples synthesized in this work showing darkened colour with increasing Ru^3+^ doping. (c) Powder XRD of Cs_3_Bi_2_I_9_ and Cs_3_Bi_2−*x*_Ru_*x*_I_9_ with *x* = 0.02, 0.04, 0.1, 0.2.

As reported previously, Cs_3_Bi_2_I_9_ crystallizes in the *P*6_3_/*mmc* space group with a lattice constant of *a* = 8.335 Å and *c* = 21.326 Å (Fig. 1a).^16^ When a portion of Bi^3+^ sites in Cs_3_Bi_2_I_9_ is substituted with Ru^3+^, the colour of the crystal powder changes from bright to dark red with the increasing doping level (Fig. 1b). The darkened colour is indicative of narrowed bandgap, as more visible light is absorbed by the compound. The PXRD data of Cs_3_Bi_2−*x*_Ru_*x*_I_9_ with *x* = 0, 0.02, 0.04, 0.1 and 0.2 are given in Fig. 1c (also amplified in Fig. S1†), from which one can see that at low doping levels, the XRD patterns exhibit no obvious change from the original Cs_3_Bi_2_I_9_ spectrum, matching well the standard profile of JCPDS no. 39-1346. This suggests the lattice structure of Cs_3_Bi_2_I_9_ is not observably affected by low levels of Ru^3+^ substitution, likely due to the smaller atomic radius of Ru^3+^ than Bi^3+^ (Fig. 1a). However, at a higher level of substitution (*x* = 0.2), more broadened brag peaks can be observed at 26°, 28°, 31°and 44°, which could be resulted from possible structural corruption, as evidenced by morphological changes from the scanning electron microscopy (SEM) characterization (Fig. S2†) and that none of these peaks is assignable to any raw materials.

**Fig. 2:**
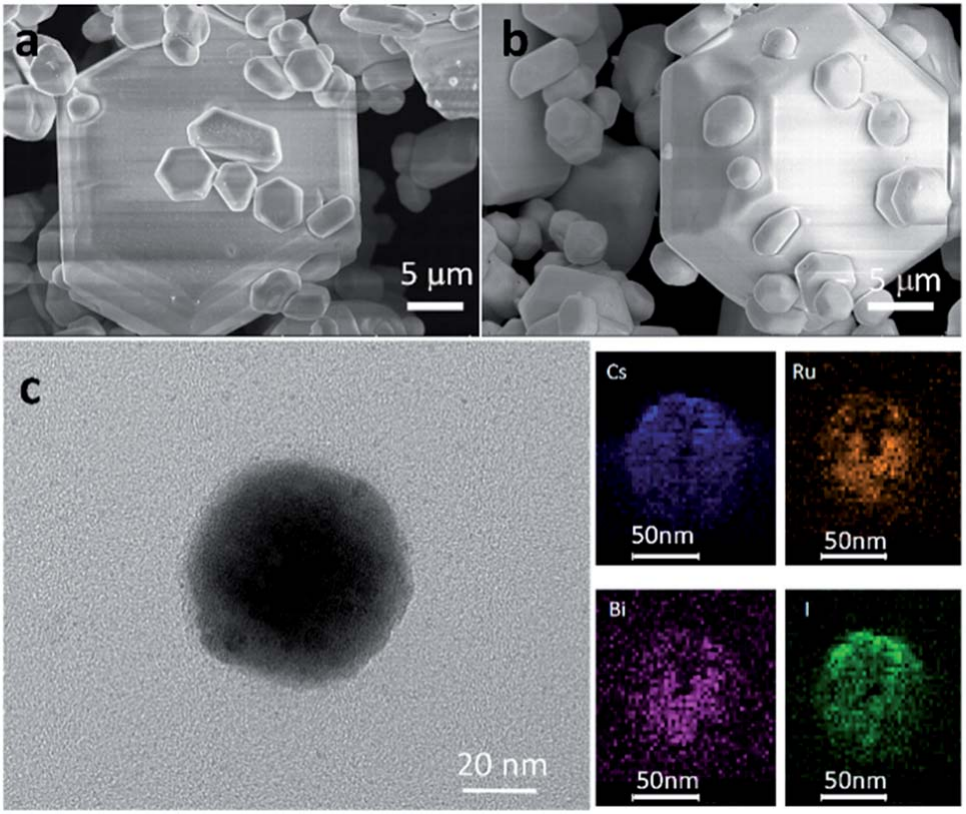
(a) and (b) SEM images of Cs_3_Bi_2_I_9_ and Cs_3_Bi_1.9_Ru_0.1_I_9_, respectively. (c) TEM image of Cs_3_Bi_1.9_Ru_0.1_I_9_ and the corresponding elemental mapping of Cs, Bi, Ru, and I.

Fig. 2a and b present the characteristic SEM images of the as-prepared Cs_3_Bi_2_I_9_ and Cs_3_Bi_1.9_Ru_0.1_I_9_ microcrystals, respectively. Particles of various size ranging from a few to hundreds of microns can be found throughout the samples. While small particles exhibit more irregular crystal shapes, those larger ones are typically hexagonal. Simultaneously, energy-dispersive X-ray spectroscopy (EDX) measurements were taken to verify the existence of Ru^3+^ in the doped samples, all showing prominent Ru peaks (Fig. S3†). Fig. 2c presents the TEM image of a small particle of Cs_3_Bi_1.9_Ru_0.1_I_9_, with the corresponding elemental mapping clearly showing that atoms of Bi and Ru are homogeneously distributed within the crystal.

**Fig. 3:**
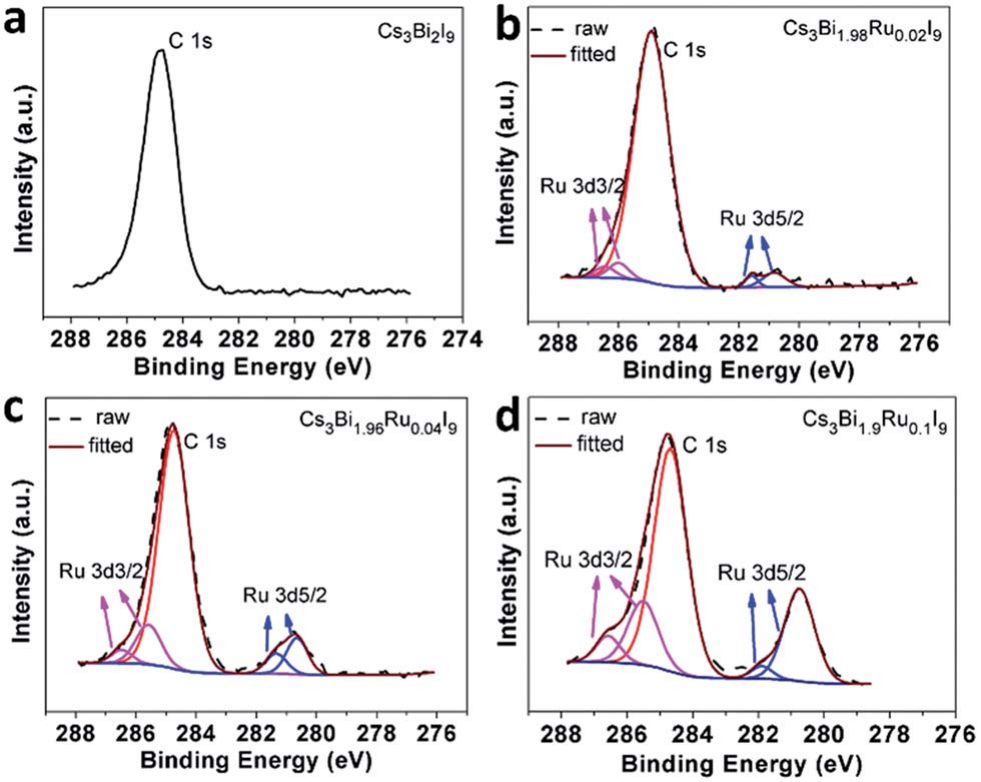
XPS spectra of Ru^3+^ for Cs_3_Bi_2−*x*_Ru_*x*_I_9_ showing the increase of peak intensity with increasing doping levels. (a) *x* = 0; (b) *x* = 0.02; (c) *x* = 0.04; (d) *x* = 0.1.

XPS analysis of surface atomic chemical states reveals all Cs, Bi, I elements for Cs_3_Bi_2_I_9_ and the additional Ru element for Cs_3_Bi_2−*x*_Ru_*x*_I_9_ (Fig. 3 and S4†). No obvious shift of chemical states is observable for Cs, Bi, and I elements upon Ru-doping. There is a carbon peak at 284.9 eV in all spectra of tested samples, attributable to atmospheric carbon introduced during the sample preparation and testing process. Neighboring to the C peak, the Ru 3d peaks are composed of two doublets, assignable to Ru 3d_3/2_ centered at around 286.0 eV and Ru 3d_5/2_ at around 281.0 eV. The XPS analysis not only proves the successful doping of Ru into Cs_3_Bi_2_I_9_, but also confirms its trivalent chemical state. In addition, the intensity of the Ru 3d_5/2_ doublet increases monotonically with the doping level. To further quantify the actual doping concentration of Ru^3+^ in replacement of Bi^3+^ in different Cs_3_Bi_2−*x*_Ru_*x*_I_9_ samples, elemental analysis by Inductively Coupled Plasma-Atomic Emission Spectrometry (ICP-AES) was carried out on the obtained crystals and the results are listed in the Table S1.† For a theoretical doping concentration of 0, 1%, 2% and 5% based on the molar ratio of added RuCl_3_ to BiCl_3_, the corresponding ICP-AES reading is 0, 0.9%, 1.9% and 4.3%, respectively. The discrepancy between the theoretical and actual Ru-doping levels could be caused by both incomplete reaction and weighting errors, but overall a good correlation between the two can be seen as long as the doping level isn't too high (>5%). For the purpose of convenience and clarification, we use the theoretical doping level to label all samples throughout this study.

### 3.2 Electronic and optical properties

**Fig. 4:**
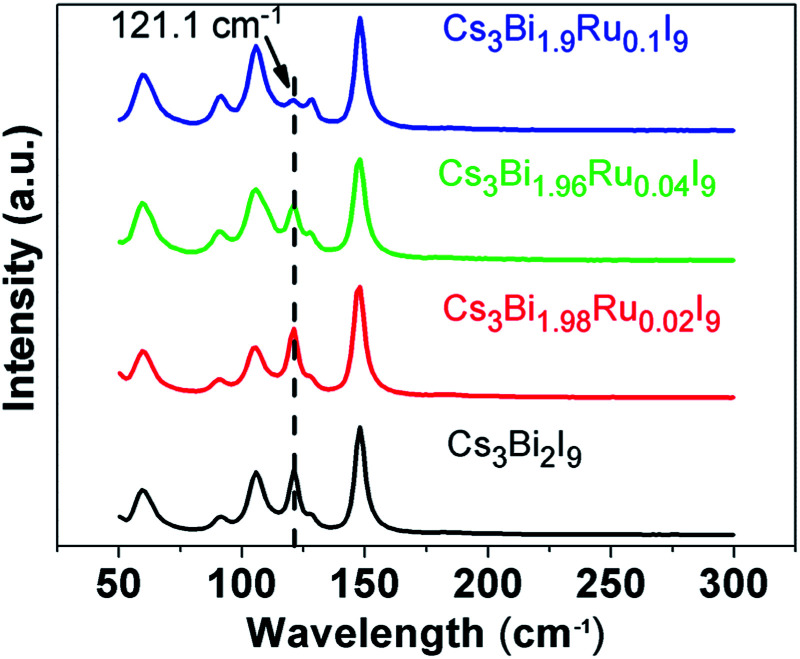
Raman spectra of the Cs_3_Bi_2−*x*_Ru_*x*_I_9_ (*x* = 0, 0.02, 0.04, and 0.1) samples.

Raman spectra were taken to illustrate the evolution of chemical bonding and lattice configuration inside the Cs_3_Bi_2−*x*_Ru_*x*_I_9_ crystals with increasing Ru^3+^ doping (Fig. 4). Within the wavenumber range from 50 cm^−1^ to 2000 cm^−1^, the obtained spectrum for each compound at room temperature reveals similar crystal structure. No peaks were found above 200 cm^−1^, and all compounds have six major peaks at the same positions without apparent peak shift. The position of these six peaks is in good agreement with what reported for Cs_3_Bi_2_I_9_ before,^26,32^ where the three peaks at higher wave number (high energy peaks) are resulted from symmetric and asymmetric stretches of the terminal Bi–I bonds in the [Bi_2_I_9_]^3−^ anion, and the middle two peaks at 105.8 cm^−1^ and 91.6 cm^−1^ are caused by symmetric and asymmetric stretches of the bridging Bi–I bonds, respectively.^33^ On the far left side, the low energy peak at 59.4 cm^−1^ represents the bending modes of the Bi–I bonds. A prominent trend observed from the Raman spectra is the gradual disappearance of the antisymmetric peak at 121.1 cm^−1^ with the increasing Ru^3+^ doping. Since both of the atomic mass and radius of Ru is smaller than Bi, one of the asymmetric stretches associated with it is likely to induce less inelastic scattering of the laser light, resulting in the weakened antisymmetric mode. As the peak at 121.1 cm^−1^ represents one of the two terminal Bi–I asymmetric stretches, this indicates the replacement of Bi with Ru has higher impact on the terminal Bi–I stretches than on the bridging B–I stretches, owing to the stronger force constants of the terminal Bi–I bonds as the bridging I atoms share their bonds with two Bi atoms.^32^ The doping of Ru^3+^ in replacement of Bi^3+^ further causes local lattice distortions, as indicated by the Raman spectral evolution, and creates new defect states as well as enhanced electron–phonon coupling, both enabling to trap excitons upon irradiated absorption.

**Fig. 5:**
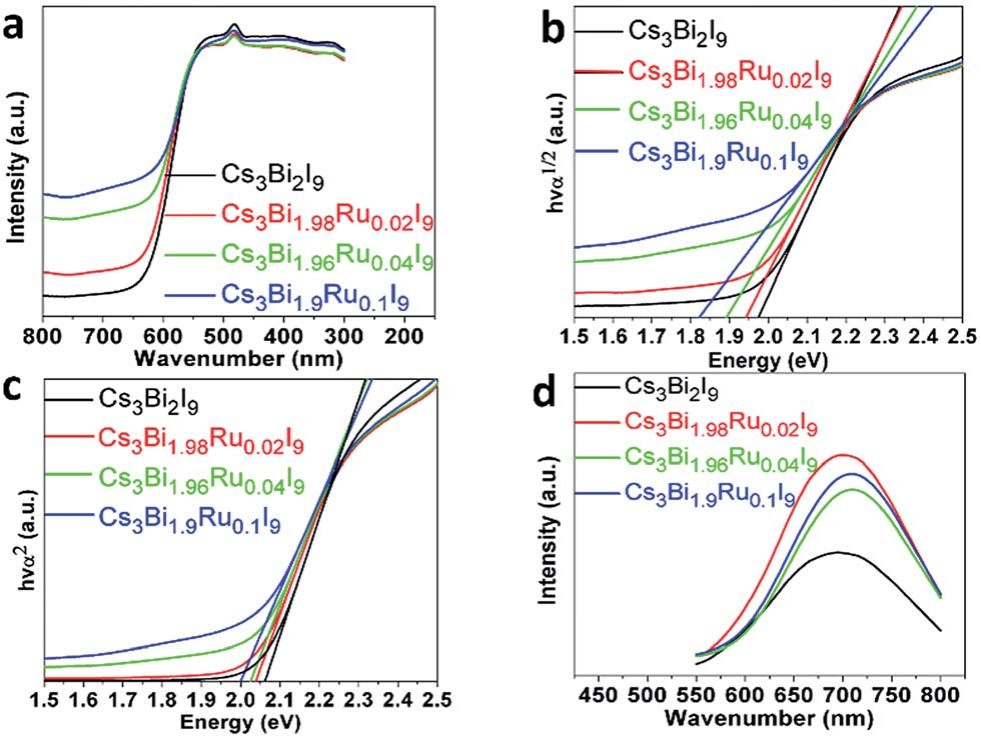
(a) The UV-Vis spectra of Cs_3_Bi_2−*x*_Ru_*x*_I_9._ (b) The direct Tauc plots of Cs_3_Bi_2−*x*_Ru_*x*_I_9_. (c) The indirect Tauc plots of Cs_3_Bi_*x*_Ru_2−*x*_I_9_. (d) The normalized PL spectra of Cs_3_Bi_2−*x*_Ru_*x*_I_9_.

As shown in the optical images in Fig. 1b, the color of the compounds turns darker as the doping amount of Ru^3+^ increases. To further quantify the light absorption and optical bandgaps of the Cs_3_Bi_2−*x*_Ru_*x*_I_9_ compounds with various Ru^3+^ doping, UV-Vis diffuse reflectance spectra were taken as shown in Fig. 5a. All acquired UV-Vis spectra display similar curves with a stronger absorption observed with the increasing Ru^3+^ amount. For all samples, the absorption starts from around 630 nm with an additional peak observed near 490 nm, corresponding to the *n* = 1 excitonic transition as seen previously.^34^ From the UV spectra, the optical bandgap can be estimated according to the equation below:

1 [*F*(*R*)*hν*]^*n*^ = *A*(*hν* − *E*_*g*_)

where *hν* is the photon energy, *A* is a proportional constant, *E*_g_ is the value of the band gap, *n* = 2 for a direct transition or 1/2 for an indirect transition, and *F*(*R*) is the Kubelka–Munk function. By linearly extrapolating [*F*(*R*)*hν*]^*n*^ = 0, indirect optical bandgaps from 1.98 eV to 1.81 eV (Fig. 5b) and direct bandgaps from 2.07 eV to 1.98 eV (Fig. 5c) can be deduced for Cs_3_Bi_2−*x*_Ru_*x*_I_9_ with increasing Ru^3+^ doping. The bandgap value of pristine Cs_3_Bi_2_I_9_ is in good agreement with previous reports,^34–36^ and the shift of bandgaps is consistent with the optical color change observed for these compounds.

To better understand the radiative exciton recombination of the Ru-doped Cs_3_Bi_2_I_9_ compounds, photoluminescence (PL) measurements were performed (Fig. 5d). All powder samples exhibit relatively broad PL emissions in the wavelength range of 500–900 nm, with the unsubstituted Cs_3_Bi_2_I_9_ showing a similar spectrum to previous literature reports.^12,27^ The broad PL emission is well-known characteristic of the defect halide perovskites such as A_3_M^III^_2_□X_9_ due to the strong electron-phonon coupling and defect-mediated recombination, which is evidenced by the observed high emission wavelength (∼700 nm) in contrast to the absorption edge (∼600 nm). In addition, the electron-phonon interaction causes local distortion of the lattice, generating so-called polarons to couple with carriers and increase their effective mass, and therefore severely broaden the optical spectra.^26^ Both of the lattice defects and polarons enable to trap excitons, intercepting the radiative recombination. With the Ru^3+^ doping, all emission peaks of Cs_3_Bi_2−*x*_Ru_*x*_I_9_ display a higher intensity and a slight red-shift towards higher wavelength, which further indicates the emission centers belong to some shallow defect-type states, rather than the intrinsic band-edge states.^12,37^ The red-shift of PL emissions (from 696 to 706 nm) with increasing Ru^3+^ doping resonates with the narrowed bandgaps as observed in the UV-Vis studies, and might also indicate the defect states become relatively deeper with the increased doping level. This argument is further supported by the fact that the intensity of PL emissions does not linearly increase with the doping level of Ru^3+^, with the highest intensity observed for Cs_3_Bi_1.98_Ru_0.02_I_9_, and then decreased thereafter. Similar phenomena of PL decreasing with increasing dopant has also been seen for In-alloyed Cs_2_AgBiBr_6_ (ref. 38) and Mn-doped Cs_2_AgInCl_6_ (ref. 39) samples.

**Fig. 6:**
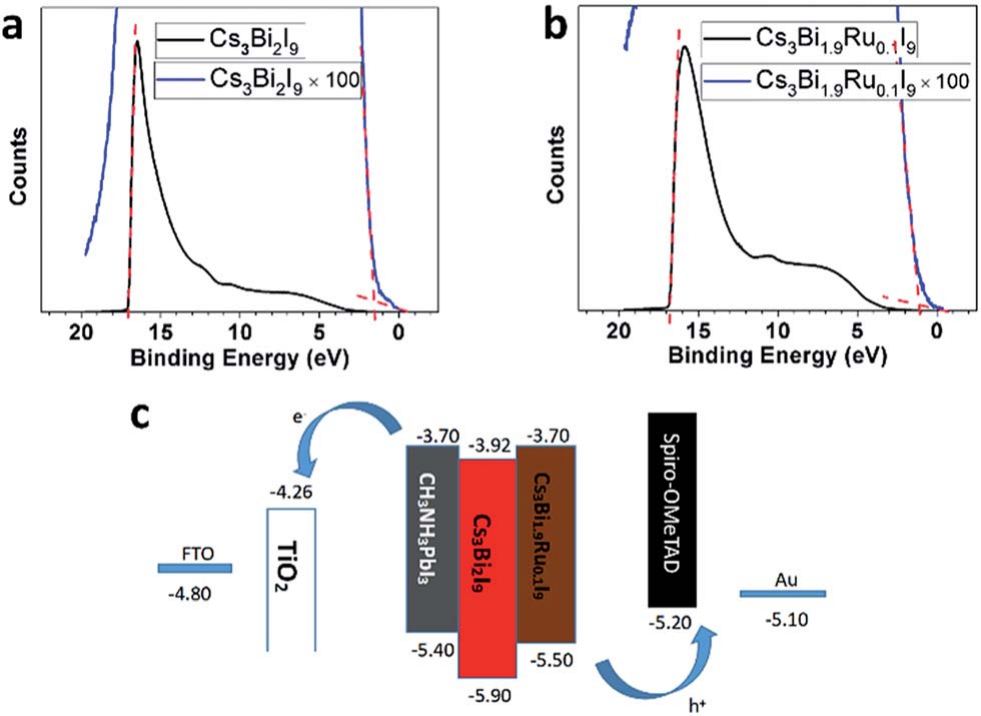
UPS spectra of (a) Cs_3_Bi_2_I_9_ and (b) Cs_3_Bi_1.9_Ru_0.1_I_9_. (c) Band structure of Cs_3_Bi_1.9_Ru_0.1_I_9_ and Cs_3_Bi_2_I_9_ in reference to MAPbI_3_, TiO_2_ and PEDOT:PSS.

To examine the effect of Ru-doping on modifying the band structure of Cs_3_Bi_2−*x*_Ru_*x*_I_9_, we employed Ultraviolet Photoelectron Spectroscopy (UPS) to measure the position of the valence band maximum (VBM) with respect to the vacuum energy for Cs_3_Bi_2_I_9_ and Cs_3_Bi_1.9_Ru_0.1_I_9_. Previous protocol developed by Lehner *et al.* was followed to capture and analyze the data and Au was used to calibrate the Fermi level.^19^ As shown in Fig. 6, the onset of the UPS spectrum starts from 1.70 eV for Cs_3_Bi_2_I_9_ and 1.10 eV for Cs_3_Bi_1.9_Ru_0.1_I_9_, with the corresponding cut-off binding energy being 17.02 and 16.82 eV, respectively. Therefore, by calculation the work function of Cs_3_Bi_2_I_9_ and Cs_3_Bi_1.9_Ru_0.1_I_9_ are 4.20 and 4.40 eV, and their corresponding VBMs are −5.90 and −5.50 eV, respectively. Further based on the optical bandgaps measured from the previously acquired UV-Vis spectra (1.98 eV for Cs_3_Bi_2_I_9_ and 1.80 eV for Cs_3_Bi_1.9_Ru_0.1_I_9_), we were able to deduce the conduction band minimum (CBM) for Cs_3_Bi_2_I_9_ and Cs_3_Bi_1.9_Ru_0.1_I_9_, being −3.92 and −3.70 eV respectively. The band positions of Cs_3_Bi_2_I_9_ are within the upper and lower bounds of previously reported range (−3.40 to −4.20 eV for CBM, −5.60 to −6.30 eV for VBM).^19^ What's more, by comparing Fig. 6a with Fig. 6b, the counts of photoelectrons within the valence band are higher on Cs_3_Bi_1.9_Ru_0.1_I_9_, indicative of a higher photoactivity, which is consistent with previous UV and PL results. Fig. 6c shows the column diagram for comparing the band positions of Cs_3_Bi_2_I_9_ and Cs_3_Bi_1.9_Ru_0.1_I_9_, with the classic perovskite photoabsorber (MAPbI_3_) and two commonly used hole-transportation (PEDOT:PSS) and electron-transportation (TiO_2_) materials as references. Upon doping with ∼5% Ru, the valance band position of Cs_3_Bi_1.9_Ru_0.1_I_9_ shifts up about 0.40 eV (*vs.* Cs_3_Bi_2_I_9_) with a narrowed bandgap of 1.80 eV, and the CBM is close to that of MAPbI_3_. As a result, both the narrowed bandgap and the upshift of the overall band structure might facilitate their wider optoelectronic applications.

### 3.3 Environmental and thermal stability

**Fig. 7:**
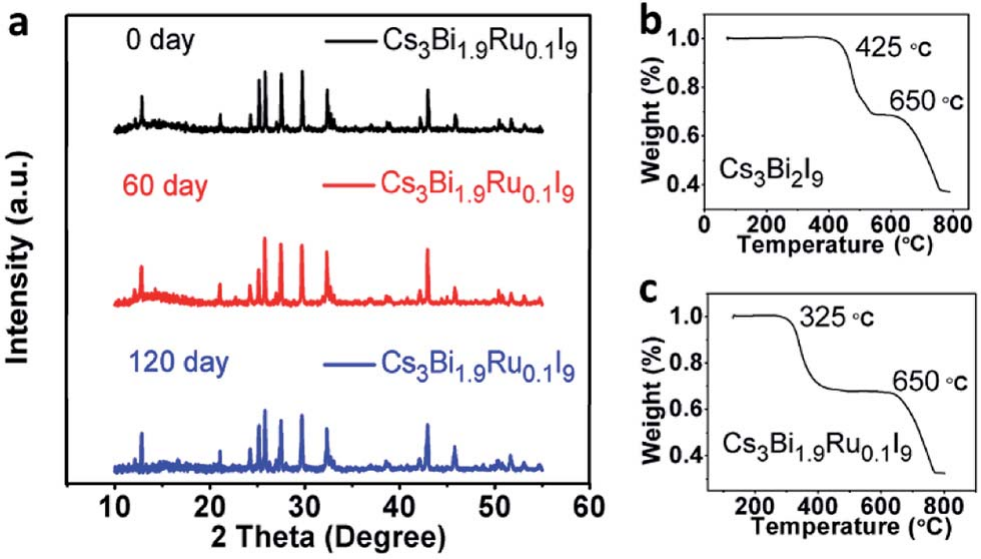
(a) PXRD spectrum of Cs_3_Bi_1.9_Ru_0.1_I_9_ after 60 days and 120 days. (b) and (c)TGA spectra of Cs_3_Bi_2_I_9_ and Cs_3_Bi_1.9_Ru_0.1_I_9_, respectively.

Stability is a key factor when evaluating perovskite materials for potential optoelectronic applications. It was reported that Cs_3_Bi_2_I_9_ is more stable than Pb-based perovskites upon exposure to the ambient atmosphere in both light and moisture conditions. To further investigate the stability of Ru-doped Cs_3_Bi_2_I_9_, we selected the Cs_3_Bi_1.9_Ru_0.1_I_9_ as an example to examine its environmental stability. XRD spectra were acquired after 60 and 120 days of full exposure to the room light and moisture (∼60% RH) of lab environments. As shown in Fig. 7a, no obvious change is observable among these spectra, demonstrating excellent environmental stability of the Ru-doped Cs_3_Bi_2_I_9_ compounds. Furthermore, we checked the thermal stability of the Cs_3_Bi_1.9_Ru_0.1_I_9_ compound under extreme conditions using the thermogravimetric analysis (TGA). A two-step weight loss was seen for both doped and un-doped Cs_3_Bi_2_I_9_, with the first weight loss around 30%, likely due to the partial sublimation of one molecule of BiI_3_ (bp ≈ 500 °C) from the parent compound.^26^ For the pristine Cs_3_Bi_2_I_9_, the first decomposition starts from 450 °C, whereas for Cs_3_Bi_1.9_Ru_0.1_I_9_ this temperature is lowered to 325 °C. A linear decrease of the first decomposition temperature was seen with the increasing Ru-doping (Fig. S5†). This provides extra evidence for the substitution of Ru^3+^ into the [Bi_2_I_9_]^3−^ bioctahedra, deteriorating its thermal stability. For all compounds, the second decomposition starts from about 650 °C, coincident with the melting point of CsI (mp ≈ 650 °C) and evaporation of the rest BiI_3_. Based on the TG curves, the decomposition products of Cs_3_Bi_1.9_Ru_0.1_I_9_ from the two weight-loss stages were further examined by XRD (Fig. S6†), showing the remained product after heating at 600 °C contains both CsI and BiI_3_, whereas that after 800 °C contains CsI and elemental Cs. It is worth to note that although the thermal stability of Cs_3_Bi_2−*x*_Ru_*x*_I_9_ deteriorates with increasing Ru-doping, they can still tolerate high temperature up to 300 °C, sufficient for most real-world applications.

## 4 Conclusions

In summary, although Cs_3_Bi_2_I_9_ has been viewed as a potential candidate for the photoabsorber of lead-free perovskite solar cells, its relatively high bandgap and deep intrinsic defect states are still the limiting factors for achieving high photoelectric conversion efficiency. In the current study, we have successfully doped Ru^3+^ into Cs_3_Bi_2_I_9_ up to 4.3% using a hydrothermal method, and obtained homogeneously dispersed crystals without damaging its crystalline structure. Optical measurements using Raman, UV-Vis and photoluminescent spectroscopy illustrated that even with a small doping amount, the optoelectronic properties of Cs_3_Bi_2−*x*_Ru_*x*_I_9_ changes significantly, including narrowed optical bandgap, induced shallow defect states, and more radiative recombination centers. UPS measurements revealed that doping of Ru^3+^ upshifts the overall band structure of Cs_3_Bi_2_I_9_ with a higher work function. Stability tests further confirmed the good environmental and thermal stability of Cs_3_Bi_2−*x*_Ru_*x*_I_9_. All these results indicate that doping of Ru^3+^ into Cs_3_Bi_2_I_9_ allows engineering the band structure by regulating its defect states as well as electronic structure. Consequently, this study shed new light on regulating the optoelectronic properties of the defect halide perovskite family through cation substitution.

## Conflicts of interest

There are no conflicts to declare.

## Supplementary Material

RA-008-C8RA04422H-s001
