# Supplementary material for: Bandgap engineering of a lead-free defect perovskite Cs3Bi2I9 through trivalent doping of Ru3+
Source: RSC Adv. 2018 Jul 18;8(45):25802–7. doi: 10.1039/c8ra04422h (PMC9082562; doi:10.1039/c8ra04422h)
Supplement: RA-008-C8RA04422H-s001 [file RA-008-C8RA04422H-s001.pdf]

Electronic Supplementary Material (ESI) for RSC Advances.
This journal is © The Royal Society of Chemistry 2018

Electronic Supplementary Information

Bandgap Engineering of a Lead-free Defect Perovskite $Cs_3Bi_2I_9$ through Trivalent Doping of Ru^{3+}

Gu, Jinyu[a,b], Yan, Gangbin[a,b], Lian, Yuebin[a,b], Mu, Qiaoqiao[a,b], Jin, Huidong[a,b], Deng, Zhao[a,b*] and Peng, Yang[a,b*]

[a] Soochow Institute for Energy and Materials Innovations, College of Physics, Optoelectronics and Energy & Collaborative Innovation Center of Suzhou Nano Science and Technology, Soochow University, Suzhou 215006, P. R. China.

[b] Key Laboratory of Advanced Carbon Materials and Wearable Energy Technologies of Jiangsu Province, Soochow University, Suzhou 215006, P. R. China.

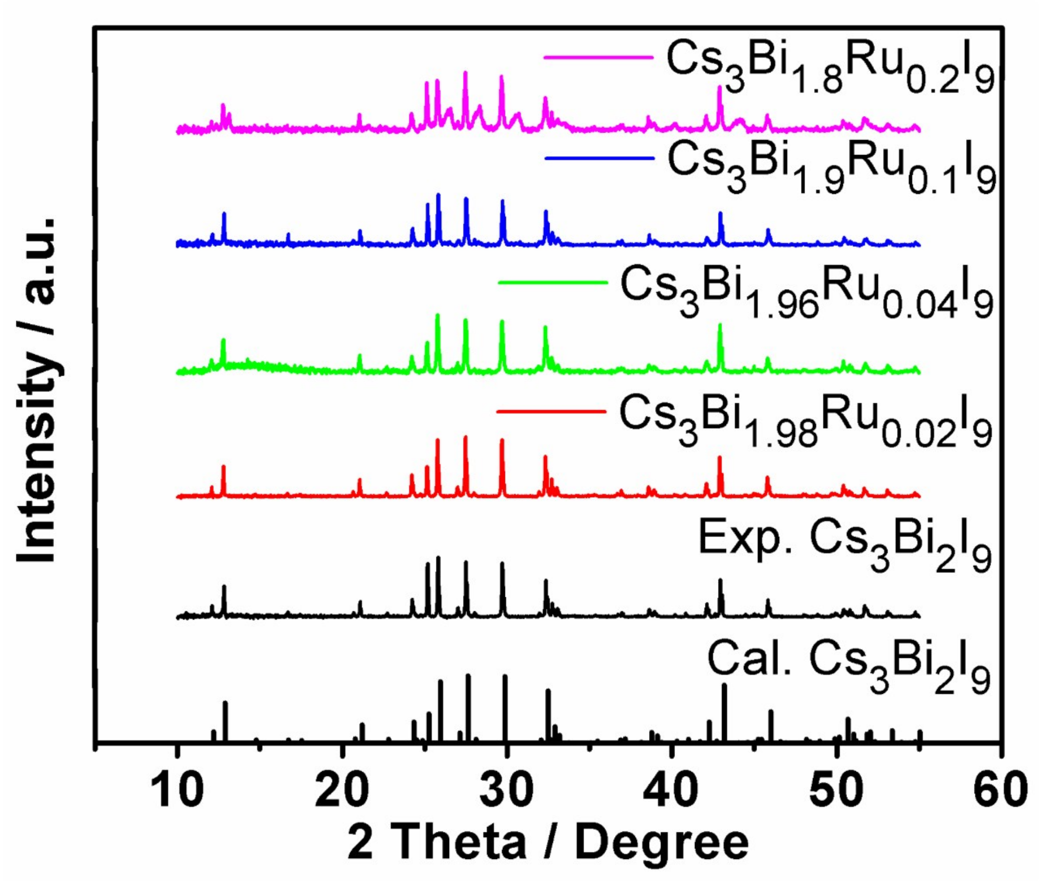

Figure S1. Powder XRD of $Cs_3Bi_2I_9$ and $Cs_3Bi_{2-x}Ru_xI_9$ with x = 0.02, 0.04, 0.1, 0.2

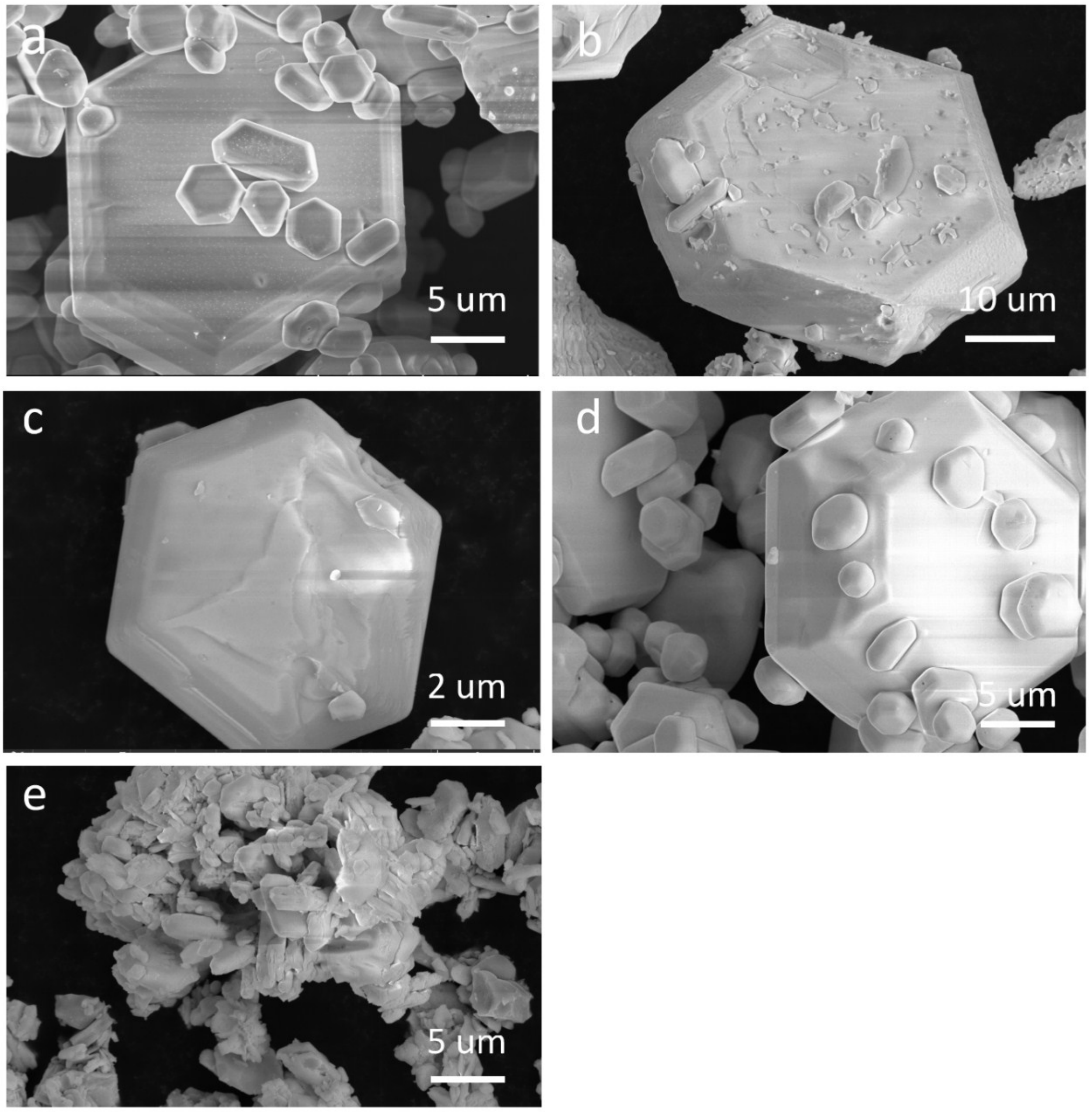

Figure S2. SEM images and of $Cs_3Bi_{2-x}Ru_xI_9$. (a) x = 0; (b) x = 0.02; (c) x=0.04; (d) x = 0.1; (e) x = 0.2.

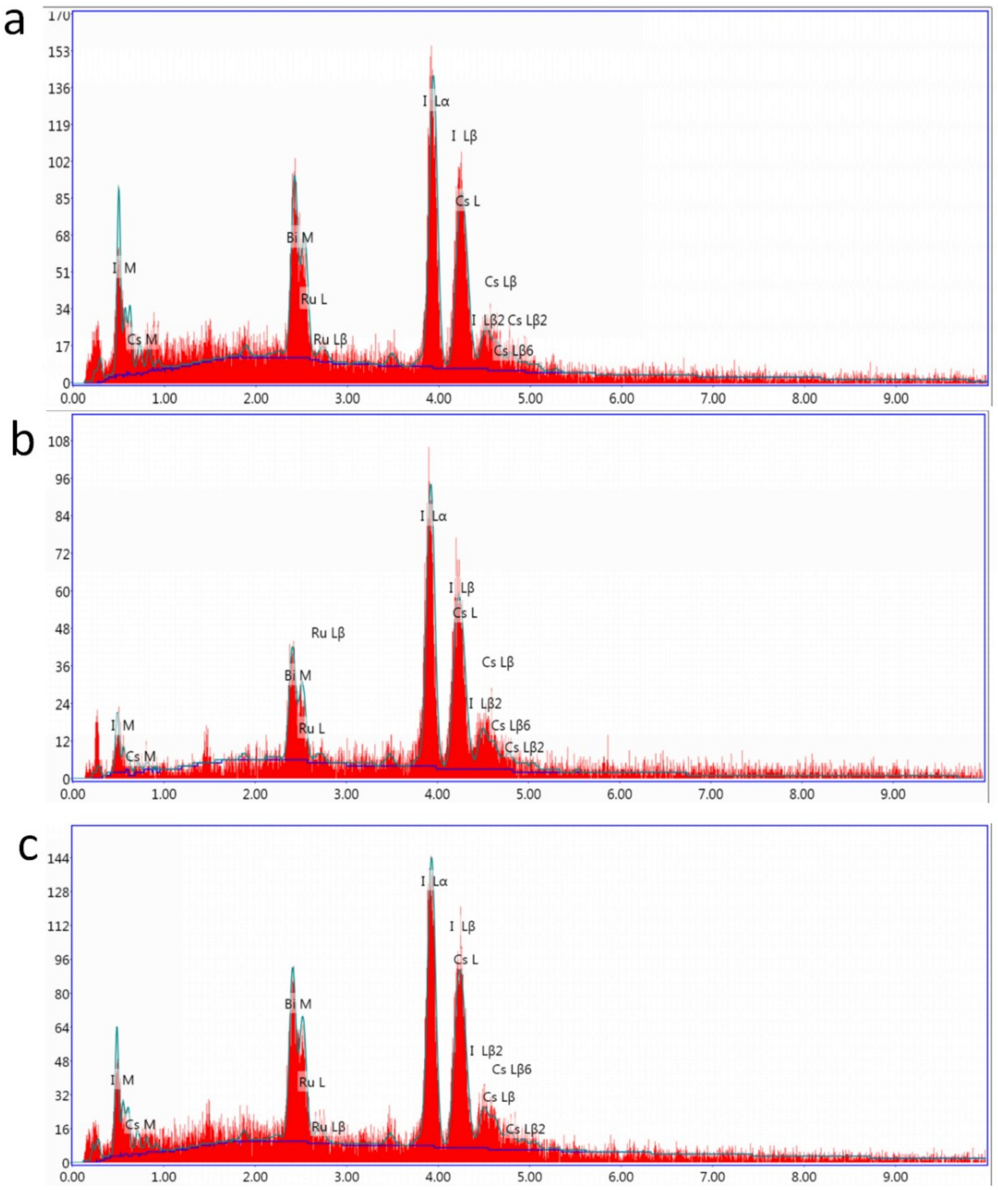

Figure S3. EDAX of $Cs_3Bi_{2-x}Ru_xI_9$. (a) x = 0.02; (b)x = 0.04; (c) x = 0.1.

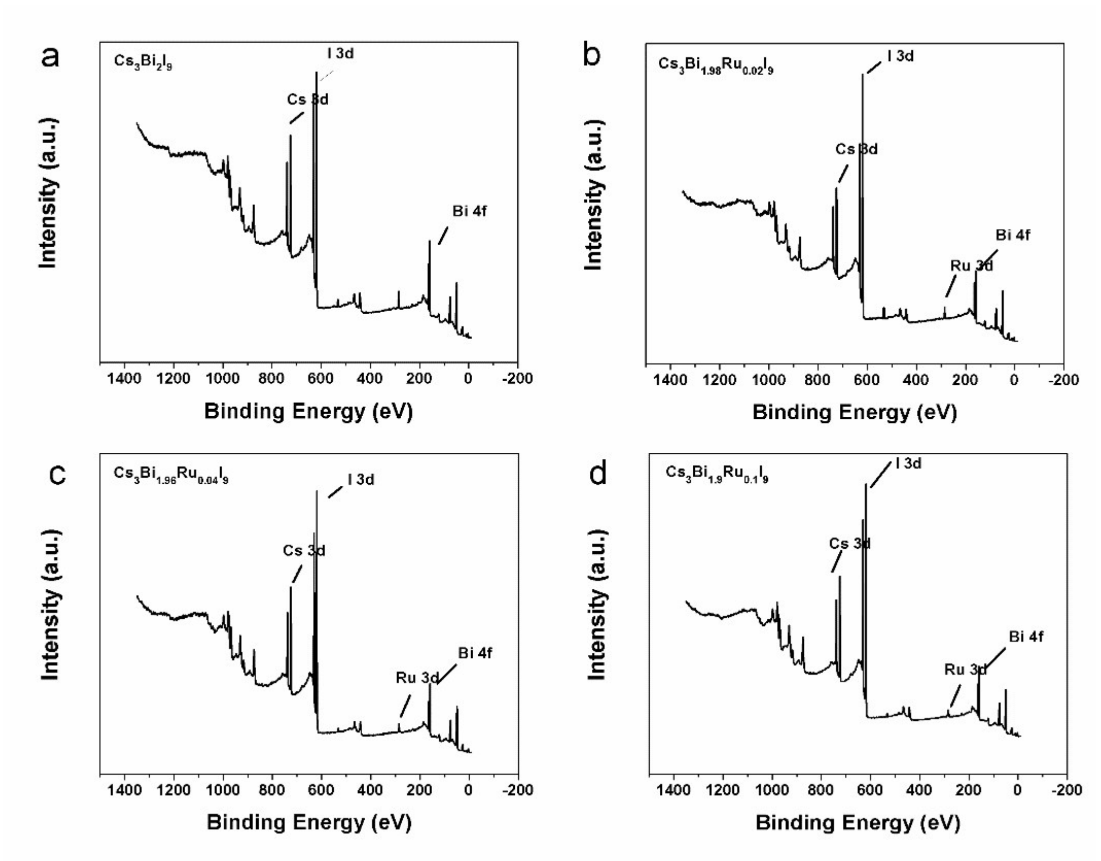

Figure S4. XPS Survey Spectra of $Cs_3Bi_2I_9$ and $Cs_3Bi_{2-x}Ru_xI_9$.
(a) $Cs_3Bi_2I_9$; (b) $Cs_3Bi_{1.98}Ru_{0.02}I_9$; (c) $Cs_3Bi_{1.96}Ru_{0.04}I_9$; (d) $Cs_3Bi_{1.9}Ru_{0.1}I_9$.

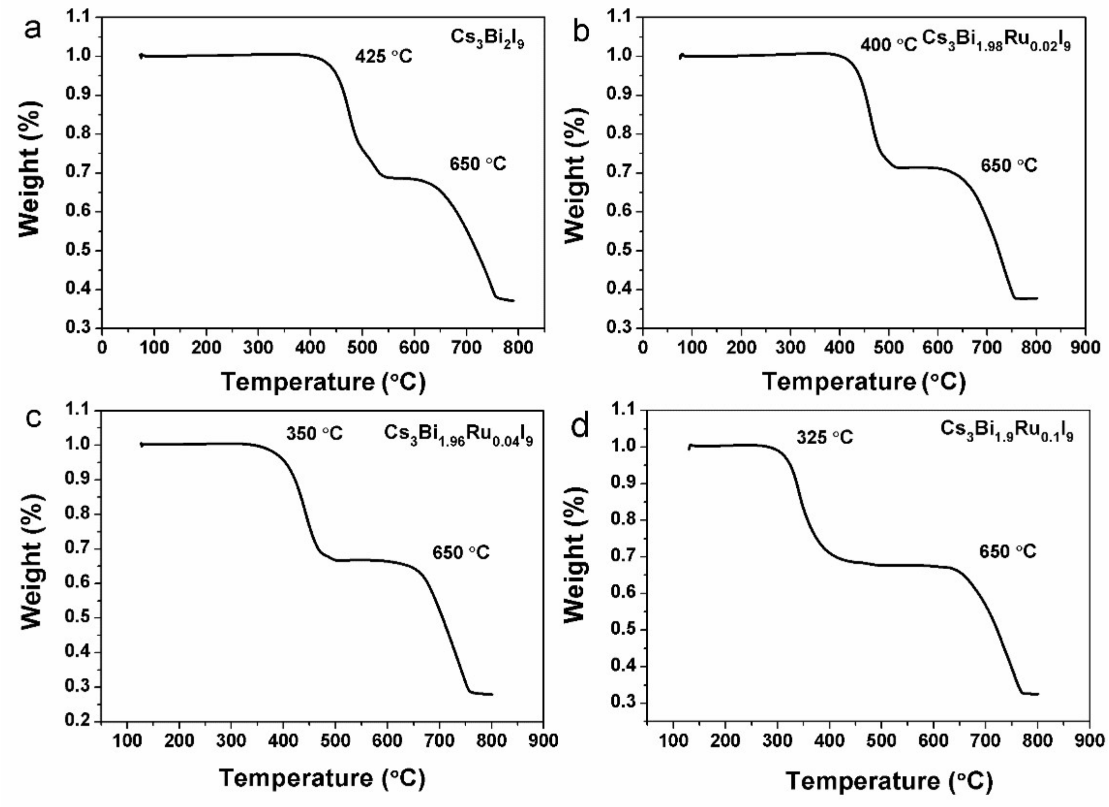

Figure S5. TG curves of $Cs_3Bi_2I_9$ and $Cs_3Bi_{2-x}Ru_xI_9$.

(a) $Cs_3Bi_2I_9$; (b) $Cs_3Bi_{1.98}Ru_{0.02}I_9$; (c) $Cs_3Bi_{1.96}Ru_{0.04}I_9$; (d) $Cs_3Bi_{1.9}Ru_{0.1}I_9$.

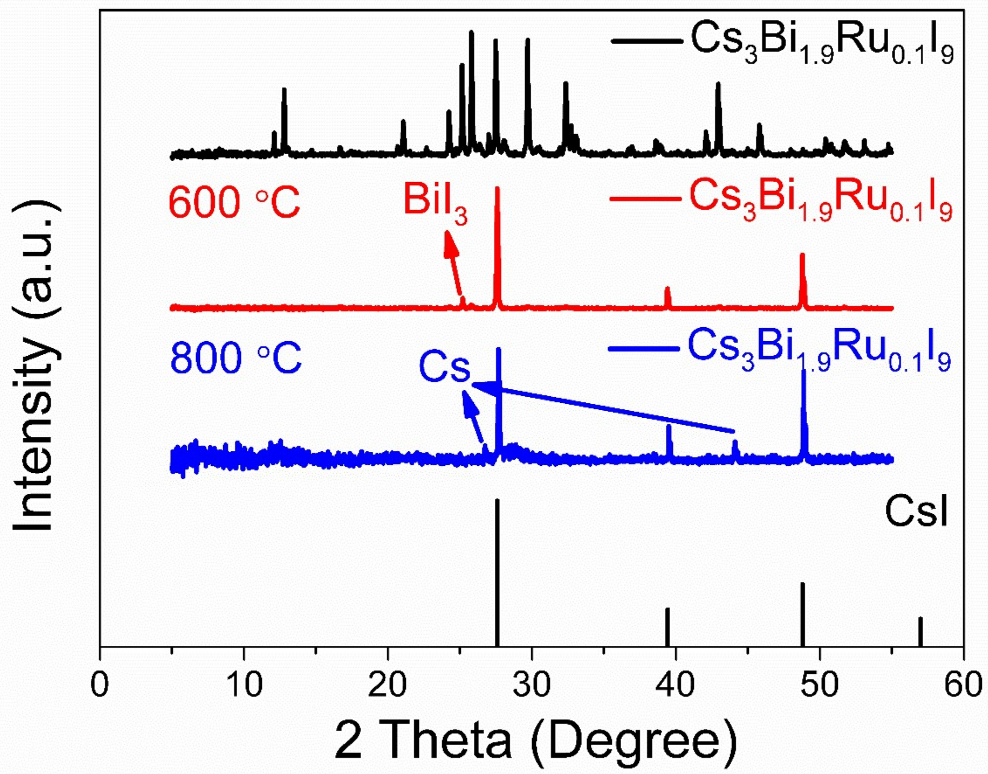

Figure S6. XRD of $Cs_3Bi_{1.9}Ru_{0.1}I_9$ after heating at 600 °C and 800 °C.

Table 1. Elemental analysis by ICP-AES for $Cs_3Bi_{2-x}Ru_xI_9$

Samples of $Cs_3Bi_{2-x}Ru_xI_9$	X = 0	X = 0.02	X = 0.04	X = 0.1
Theoretical doping level	0	1%	2%	5%
Actual doping level by ICP-AES (%)	0	0.9%	1.9%	4.3%
